# Synthesis of Multiple Bispecific Antibody Formats with Only One Single Enzyme Based on Enhanced Trypsiligase †

**DOI:** 10.3390/ijms23063144

**Published:** 2022-03-15

**Authors:** Johanna Voigt, Christoph Meyer, Frank Bordusa

**Affiliations:** Department of Naturstoffbiochemie, Institute for Biochemistry and Biotechnology, Martin-Luther-Universität Halle-Wittenberg, Kurt-Mothes-Straße 3a, 06120 Halle (Saale), Germany; johanna.hohgardt@biochemtech.uni-halle.de (J.V.); christoph.meyer@biochemtech.uni-halle.de (C.M.)

**Keywords:** bispecific antibody, Trypsiligase, click chemistry, biorthogonal chemistry, antibody engineering

## Abstract

Bispecific antibodies (bsAbs) were first developed in the 1960s and are now emerging as a leading class of immunotherapies for cancer treatment with the potential to further improve clinical efficacy and safety. Many different formats of bsAbs have been established in the last few years, mainly generated genetically. Here we report on a novel, flexible, and fast chemo–enzymatic, as well as purely enzymatic strategies, for generating bispecific antibody fragments by covalent fusion of two functional antibody Fab fragments (Fabs). For the chemo–enzymatic approach, we first modified the single Fabs site-specifically with click anchors using an enhanced Trypsiligase variant (eTl) and afterward converted the modified Fabs into the final heterodimers via click chemistry. Regarding the latter, we used the strain-promoted alkyne-azide cycloaddition (SPAAC) and inverse electron-demand Diels–Alder reaction (IEDDA) click approaches well known for their fast reaction kinetics and fewer side reactions. For applications where the non-natural linkages or hydrophobic click chemistry products might interfere, we developed two purely enzymatic alternatives enabling *C*- to *C*- and *C*- to *N*-terminal coupling of the two Fabs via a native peptide bond. This simple system could be expanded into a modular system, eliminating the need for extensive genetic engineering. The bispecific Fab fragments (bsFabs) produced here to bind the growth factors ErbB2 and ErbB3 with similar K_D_ values, such as the sole Fabs. Tested in breast cancer cell lines, we obtained biologically active bsFabs with improved properties compared to its single Fab counterparts.

## 1. Introduction

Bispecific antibodies (bsAbs) form a heterogeneous family of biological therapeutics. This fast-growing class of therapeutics holds, among others, promise mainly for the treatment of cancer and inflammatory diseases [1,2,3]. In 2020, a total of three bispecific antibodies were approved and more than 100 are being studied in clinical trials alone for the treatment of cancer [4].

In contrast to conventional monoclonal antibodies (mAbs), bsAbs simultaneously bind to two different types of antigens or, in their biparatopic form, two different epitopes on the same antigen, e. g., a cell target with a receptor on an immune cell or several targets on a cell surface to increase the cytotoxic potential and achieve improved efficacy with lower target expression [2]. In addition to full-length bsAbs, small bsAbs are of similar therapeutic interest due to the absence of the Fc-region. Better tissue penetration, the lack of Fc-mediated antibody effector functions, and easier production are only a few of the advantages of using antibody fragments [1,3].

The development of bsAbs accelerated in the 1980s with the progress of the synthesis techniques for their production. In particular, quadroma technology should be mentioned, which led to non-uniform bsAbs due to incorrect chain pairings, but nevertheless made numerous innovative bsAb formats possible [5,6,7]. One example is the small bsAb BiTE [8]. An important synthetic innovation to prevent mismatches of the heavy chains was the development of the knob-into-hole strategy [9]. At the same time, the first approaches were developed to suppress the mismatch of the light chains as well. In 2007, DVD (dual-variable-domain)-IgGs were developed that even had multiple fused antigen-binding domains [10]. Although relatively easy to generate by heterologous expression, their function can be influenced by the direct *N*- to *C*-terminal fusion of the domains. Antigen binding of the inner domain may be particularly affected. In addition, the increasing size of the bsAbs may also reduce the expression yield or homogeneity of the products [11,12,13]. Classical *C*- to *N*-terminal topology in bsAbs, therefore, requires considerable empirical control in the development process.

Posttranslational linkage of the single antigen-binding domains of mAbs or Fabs could address these limitations and simultaneously increase the flexibility regarding the individual structure of the final bsAbs/bsFabs. In addition to the classical *N*- to *C*-fusion of the antigen-binding domains, posttranslational domain coupling also allows more flexible orientations, e.g., *C*- to *C*-terminal domain linkage with free *N*-termini of the two binding domains, as is the case with native mAbs. Enzymatic coupling approaches seem to be particularly interesting for this purpose due to their inherent regio- and stereospecificity [14,15]. Nevertheless, only a few exceptions for purely enzymatic *C*- to *C*-terminal domain linkages are currently known [16,17]. In the most prominent case of the transpeptidase SortaseA (SrtA), a biorthogonal strategy was developed only with the help of a second enzyme, i.e., butelase1, using two different linker sequences, each specific for one enzyme [17]. A method that in principle only requires one enzyme was developed for transglutaminase [18]. The limitation of this approach, however, is that no human multi-chain Fabs or Abs were ligated, but instead camelidea single-chain domain antibodies were, which were also identical in their structure and thus, also specificity, and, therefore, led to the formation of monospecifics. Furthermore, in addition to the formation of the monospecific homodimers, these reactions also led to the simultaneous formation of inhomogeneous homomultimers with variable numbers of monomeric antibody domains. Chemo–enzymatic approaches have been developed as an alternative to installing a click anchor by enzyme catalysis to the proteins of interest first and subsequently coupling both moieties by click chemistry. Despite these promising strategies, there are, so far, only two enzymes with which the chemo–enzymatic generation of bsAbs (or analogs) has been initially demonstrated, i.e., SrtA and the formylglycine generating enzyme (FGE). In the case of SrtA, two full-length antibodies were chemo–enzymatically ligated, one with the strain-promoted alkyne-azide cycloaddition (SPAAC) *C*- to *C*-terminally to form a bsAb and, second, an antibody-(scFv)_2_ conjugate using the inverse electron-demand Diels–Alder reaction (IEDDA) [19]. A general disadvantage regarding the combination with click chemistry may result from the catalytically active cysteine of SrtA which was shown to lower the yield in the modification step with SPAAC linkers. In the case of FGE, the cysteine to formylglycine conversion activity of the enzyme was used to form an aldehyde tag (CXPXR) in the proteins of interest [20,21]. The resulting formylglycines were subsequently reacted in a two-step process with a biofunctionalized linker by Pictet–Spengler ligation, resulting in the heterodimeric product. Unfortunately, the enzyme needs reducing reaction conditions to be catalytically active. This should be the reason why only single-chain nanobodies instead of disulfide-bridged antibodies have been used so far for bsAbs synthesis [16].

As an alternative to SrtA and FGE, we developed Trypsiligase (Tl), a trypsin-based biocatalyst by rational enzyme design [22]. Tl recognizes the short, hydrophilic amino acid tag YRH and catalyzes peptide bond formation via reverse proteolysis [22,23]. The synthesis reactions take place after the tyrosine in the YRH-motif. During synthesis, the enzyme allows the covalent coupling of functional groups attached to the enzyme-specific, RH-containing nucleophile, such as fluorophores, toxins, drugs, or click anchors [23,24]. Recently, we generated next-generation Trypsiligases via evolutionary enzyme engineering [25]. These variants are characterized by an increased synthesis potential compared to the original Tl. Enhanced Trypiligase (eTl), representing the most efficient next-generation Trypsiligase so far, reaches product yields close to the thermodynamic maximum of the reaction type catalyzed.

While enzyme catalysis guarantees a regioselective reaction process, click chemistry opens the possibility of a flexible and modular synthesis principle including *C*- to *N*-, *C*- to *C*-terminal, and *N*- to *N*-terminal linkages of antigen-binding domains as well. Like enzyme synthesis, the click reactions are also biorthogonal and can be conducted in aqueous systems [26]. SPAAC and the IEDDA have been proven to be particularly suitable for biological applications. Both click reactions are fast, proceed entirely without or with only a few side reactions, and require only small substrate excesses [27,28]. In some cases, hydrophobic, sometimes bulky product structures may lead to undesirable problems with hydrophobicity, yield, or functionality.

In the present study, a synthesis approach for bsAbs is presented that only requires a single enzyme and can optionally be combined with click chemistry. It enables simple and rapid post-translational domain shuffling of antigen-binding domains to assess the optimal architecture of the bsAb. The function of the approach was evaluated using the example of the synthesis of bsFabs consisting of the antigen-binding domains of the growth factors ErbB2 and ErbB3. Both domains were covalently linked in a flexible orientation, purely enzyme-catalyzed or chemo–enzymatically with the enzymatic linkage of click anchors and downstream click chemistry. All bsFabs bind with similar K_D_ values as the individual Fabs. Tests in breast cancer cell lines demonstrated improved properties in the biological activity of the bsFabs compared to their single Fab counterparts.

## 2. Results and Discussion

The concept of posttranslational bsAbs assembling via eTl was investigated by fusing the antigen-binding moieties of two distinct Fabs, i.e., anti-ErbB2- and anti-ErB3-Fab. Both Fabs target epitopes on growth factor receptors of the EGFR family. ErbB2 is abundant in many tissue tumors, such as breast cancer [29,30]. At the same time, ErbB3 is the preferred interaction partner of ErbB2. Once they interact, they form the most robust signal within this family of receptors [31,32]. The ErbB2-specific Fab used is derived from the well-known antibody Trastuzumab, whereas the anti-ErbB3 counterpart is derived from CDX3379, a therapeutic antibody from Celldex therapeutics [33,34].

**Scheme 1:**
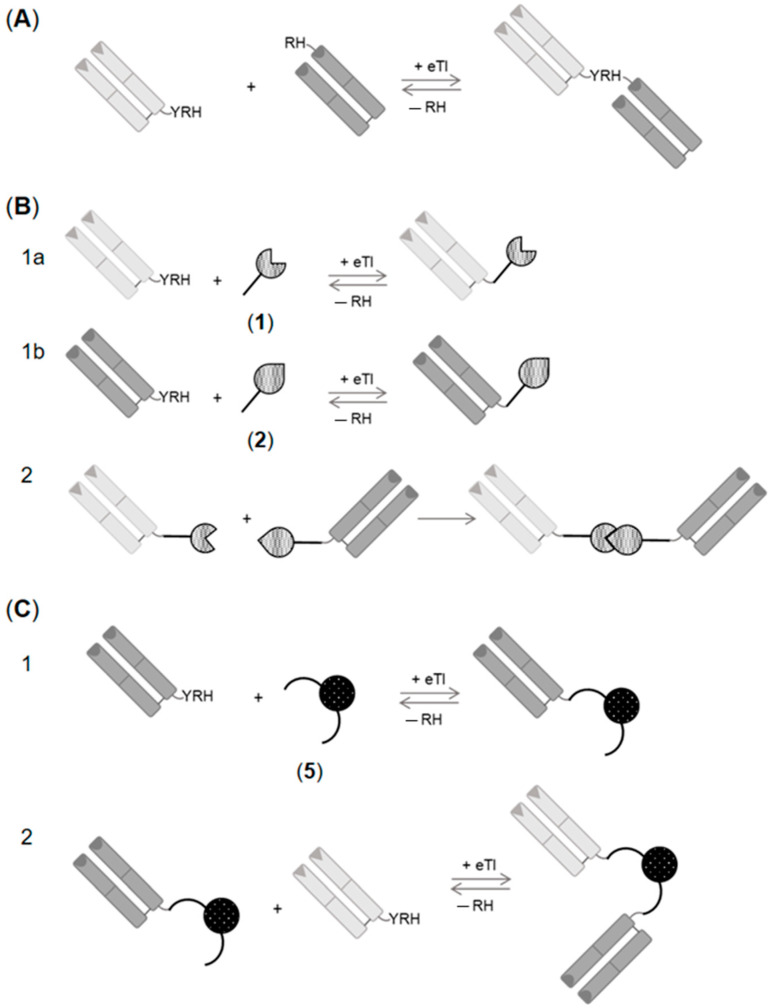
Synthesis and structure of anti-ErbB2-anti-Erb3-bsFab formats. (**A**) Enzymatic synthesis of *C*- to *N*-linked anti-ErbB3-YRH-anti-ErbB2-bsFab via eTl-catalysis from anti-ErbB3-Fab-YRH and anti-ErbB2-RH-Fab; (**B**) Chemo–enzymatic synthesis of *C*- to *C*-linked anti-ErbB3-anti-ErbB2-bsFab via eTl-mediated coupling of click anchors to both Fabs (reaction 1a and 1b), followed by click coupling of the purified intermediate Fab products (reaction 2); (**C**) Enzymatic synthesis of *C*- to *C*-linked anti-ErbB3-anti-ErbB2-bsFab via a two-step eTl-catalysis initiated by the enzymatic coupling of anti-ErbB2-Fab-YRH to linker **5**, followed by a second enzymatic coupling of the purified intermediate to anti-ErbB3-Fab-YRH. Light grey Fab: anti-ErbB3-Fab-YRH, dark grey Fab: anti-ErbB2-Fab; eTl: enhanced Trypsiligase.

**Figure 1:**
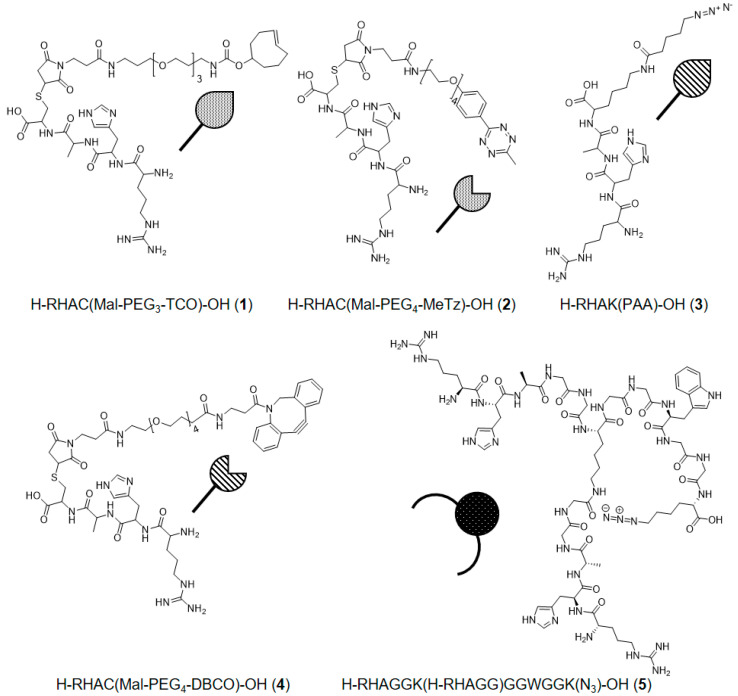
Structures of click linkers used. H-RHAC(Mal-PEG_3_-TCO)-OH (**1**) and H-RHAC(Mal-PEG_4_-MeTz)-OH (**2**) were used for IEDDA; H-RHAK(PSA)-OH (**3**) and H-RHAC(Mal-PEG_4_-DBCO)-OH (**4**) were used for SPAAC; H-RHAGGK(H-RHAGG)GGWGGK(N_3_)-OH (**5**) was used for enzymatic *C*- to *C*-terminal Fab coupling. Mal: maleiimide, TCO: *trans*-cyclooctene, MeTz: methyltetrazine, PAA: pentanoic acid azide, DBCO: dibenzocyclooctyne.

Initially, the Fabs were equipped with a nucleotide sequence encoding either the *C*-terminal YRH- or *N*-terminal RH-motif on the genomic level. Both enzyme recognition sequences were inserted into the respective termini of the heavy chain of the two Fabs. Biosynthesis and purification of the constructs were performed according to established protocols [23,33]. Final assembly of bsFabs was evaluated either by direct coupling of a *C*-terminally tagged anti-ErbB3-Fab-YRH with the *N*-terminally modified anti-ErbB2-RH-Fab via eTl (Scheme 1A) or indirectly via eTl-coupling of click anchors to the anti-ErbB2- and anti-ErbB3-Fabs followed by click chemistry-mediated bsFab formation (Scheme 1B). Direct coupling via eTl according to Scheme 1A resulted in a *C*- to *N*- linkage of anti-ErbB3- and anti-ErbB2-Fab. On the contrary, *C*- to *C*-terminal linker structure was realized by click chemistry due to the eTl-coupling of the click anchors to the *C*-terminus of the two Fabs. The individual structure of the IEDDA and SPAAC based click linkers are shown in Figure 1. Previous studies showed that Tl also catalyzes the attachment of artificial functionalities to the *N*-terminus of proteins with high yields [24]. Finally, we tried to assemble a *C*- to *C*-linked anti-ErbB3-anti-ErbB2-bsFab by a purely enzymatic approach using the branched linker **5** (Figure 1) for Fab-coupling featuring two eTl-specific *N*-termini (Scheme 1C). *C*- to *C*-linkage was achieved by a two-step enzymatic reaction, ligating the first linker **5** to anti-ErbB2-Fab-YRH followed by the coupling of anti-ErbB3-Fab-YRH. The central lysine moiety in **5** allows the incorporation of a third functionality in addition to the two enzyme recognition sites. In our case, we used an azide to enable click chemistry with spectroscopic labels.

### 2.1. Enzymatic Synthesis of C- to N-Linked Anti-ErbB3-Anti-ErbB2-bsFab

**Figure 2:**
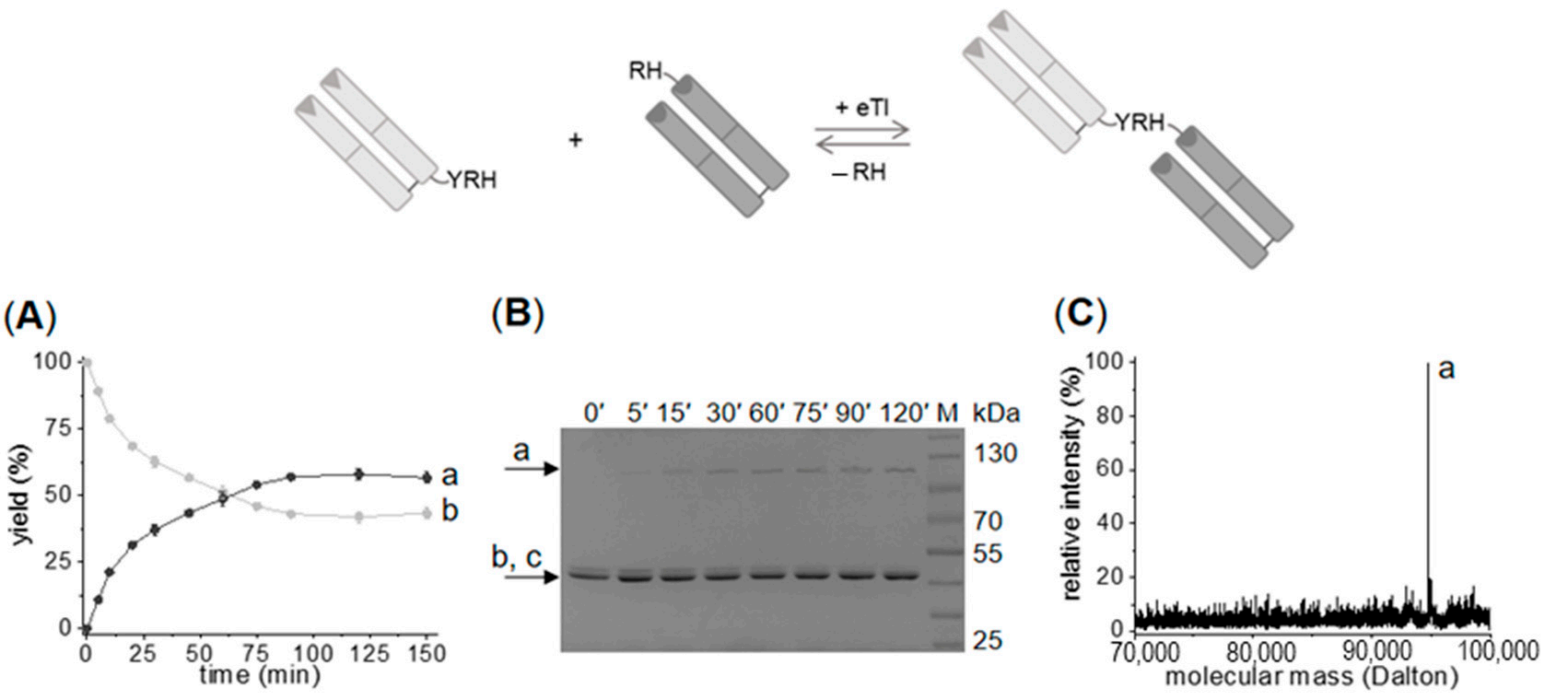
Synthesis of *C*- to *N*-terminal conjugated bsFab via direct eTl-coupling of anti-ErbB3-Fab-YRH and anti-ErbB2-RH-Fab. (**A**) Kinetics of product formation with reaction yields of about 60%, a: anti-ErbB3-YRH-anti-ErbB2-bsFab, b: anti-ErbB2-RH-Fab; (**B**) Time-resolved SDS-PAGE analysis of coupling reaction, c: anti-ErbB3-Fab-YRH, M: molecular marker; (**C**) MS analysis of the final bsFab product after purification by SEC, anti-ErbB3-YRH-anti-ErbB2-bsFab M_calcd_: 94,804 Da, M_found_: 94,805 Da. Light grey Fab: anti-ErbB3-Fab-YRH, dark grey Fab: anti-ErbB2-Fab-RH; eTl: enhanced Trypsiligase. Reaction conditions: 20 µM anti-ErbB2-RH-Fab, 100 µM anti-ErbB3-Fab-YRH, 2.5 µM eTl, 50 µM ZnCl_2_, 100 mM HEPES/NaOH, pH 7.8, 100 mM NaCl, 10 mM CaCl_2_.

The eTl-catalyzed direct coupling of anti-ErbB3-Fab-YRH and anti-ErbB2-RH-Fab, leading to the respective *C*- to *N*-linked bsFab conjugate, allows a simple one-step reaction regime, with a maximum yield of 60% of the bsFab product after reaction times of about 90 min at the reaction conditions used (Figure 2). After isolation by size exclusion chromatography (SEC), a single homogeneous bsFab conjugate was verified by LC-MS (Figure 2C), which was subsequently tested for biological functionality (Section 2.3). It should be mentioned that besides the main product and starting Fab substrates, only one further reaction product was found corresponding to the anti-ErbB3-Fab-Y-OH species in which the last two amino acid residues (RH) of the recognition sequence were missing. This indicated a certain enzymatic hydrolysis activity by eTl at the Tyr-Arg site, which is, however, usually negligible for this enzyme. The slightly reduced product yields indicated somewhat higher hydrolysis rates which may be due to limited accessibility of the enzyme recognition sequence at the anti-ErbB2-RH-Fab.

### 2.2. Generation of C- to C-Linked Anti-ErbB2-Anti-ErbB3-bsFab

Posttranslational synthesis of *C*- to *C*-linked anti-ErbB2-anti-ErbB3-bsFab was evaluated by chemo–enzymatic and purely enzymatic approaches as well. Both are two-step reaction processes in which the Fab substrates were first site-specifically modified and then converted into the final bsFabs.

#### 2.2.1. Chemo–Enzymatic Synthesis of *C*- to *C*-Linked Anti-ErbB2-Anti-ErbB3-bsFab

Chemo–enzymatic assembly of *C*- to *C*-linked anti-ErbB2-anti-ErbB3-bsFab begins with the site-specific coupling of click anchors to the starting Fabs by eTl-catalysis. For IEDDA click chemistry, the *trans*-cyclooctene (TCO) based click linker **1** was enzymatically coupled to anti-ErbB2-Fab-YRH, while the methyltetrazine (MeTz) anchor **2** was linked to anti-ErbB3-Fab-YRH. In the case of SPAAC chemistry (Figure S1), pentanoic acid azide (PAA) (compound **3**) instead of MeTz was used, and dibenzocyclooctyne (DBCO) (compound **4**) instead of TCO. Regardless of the nature of the click reagent, similar reaction conditions were used for all couplings.

**Figure 3:**
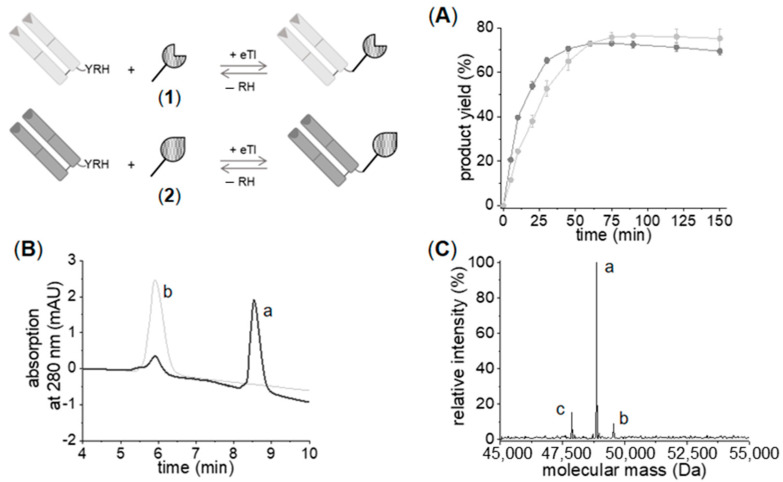
Coupling of anti-ErbB2-Fab-YRH and anti-ErbB3-Fab-YRH to click linkers **1** and **2**, respectively, via eTl-catalysis. (**A**) Reaction kinetics with maximum product yields higher than 72%, light grey line: anti-ErbB2-Fab-TCO, grey line: anti-ErbB3-Fab-MeTz; (**B**) HPLC analysis of the reaction mixture of anti-ErbB2-Fab-YRH with TCO click linker **1** at 0 min (light grey) and 75 min (dark grey) reaction times; (**C**) Mass spectrogram of the reaction mixture of anti-ErbB2-Fab-YRH with TCO click linker **1** after 75 min reaction time and after separation of excess click anchor via affinity chromatography (a: anti-ErbB2-Fab-TCO M_calcd_: 48,860 Da, M_found_: 48,861 Da, b: anti-ErbB2-Fab-YRH M_calcd_: 49,548 Da, M_found_: 49,549 Da, c: anti-ErbB2-Fab-Y-OH M_calcd_: 47,870 Da, M_found_: 47,871 Da). Light grey Fab: anti-ErbB3-Fab-YRH, dark grey Fab: anti-ErbB2-Fab-YRH; eTl: enhanced Trypsiligase. Reaction conditions: 100 µM anti-ErbB2-Fab-YRH and anti-ErbB3-Fab-YRH, 500 µM click linker **1** and **2**, 10 µM eTl, 100 µM ZnCl_2_, 100 mM HEPES/NaOH, pH 7.8, 100 mM NaCl, 10 mM CaCl_2_.

The typical course of the eTl-mediated coupling reactions is shown in Figure 3 as an example of the synthesis of anti-ErbB2-Fab-TCO and anti-ErbB3-Fab-MeTz. According to Figure 3A, product yields higher than 72% were reached after approximately 75 min of reaction time. The reactions proceeded cleanly and resulted in an equilibrium between the reaction product and starting substrate at the end of the reaction (Figure 3B). Undesired by-products did not occur apart from traces of partially hydrolyzed anti-ErbB2-Y-OH (Figure 3C). With respect to former studies, such reaction processes are rather typical for eTl-catalyzed transamidation reactions [25]. Comparable yields for the SPAAC-based products, i.e., PAA and DBCO, were found in reactions with linkers **3** and **4** (Figure S1A). Following synthesis, the reaction products were isolated by affinity chromatography mainly to remove the excess click anchor substrates and eTl. The remaining quantities of Fab substrates, on the other hand, did not interfere with the further course of synthesis.

**Figure 4:**
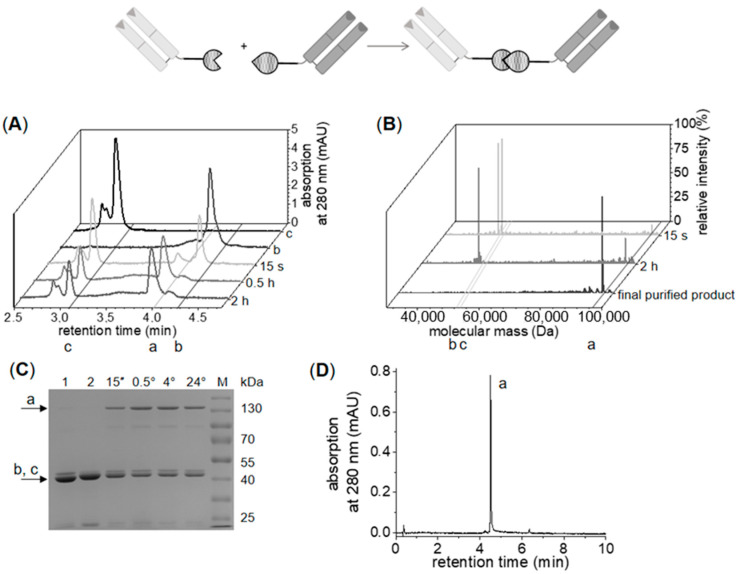
Course and analysis of the click reaction of eTl synthesized anti-ErbB3-Fab-MeTz with anti-ErbB2-Fab-TCO forming anti-ErbB3-Fab-IEDDA-anti-ErbB2-bsFab. (**A**) Time-resolved UPLC analysis of the click reaction showing a complete conversion within 0.5 h of the reaction time, a: anti-ErbB3-Fab-IEDDA-anti-ErbB2-bsFab, b: anti-ErbB3-Fab-MeTz, c: anti-ErbB2-Fab-TCO; (**B**) MS analysis of the click reaction after reaction start, 2 h reaction time and, of the final conjugation product (a: anti-ErbB3-Fab-IEDDA-anti-ErbB2-bsFab M_calcd_: 96,536 Da, M_found_: 96,537 Da, b: anti-ErbB3-Fab-MeTz M_calcd_: 47,704 Da, M_found_: 47,705 Da, c: anti-ErbB2-Fab-TCO M_calcd_: 48,860 Da, M_found_: 48,861 Da); (**C**) Time-resolved SDS-PAGE analysis of the click reaction (1: anti-ErbB3-Fab-MeTz, 2: anti-ErbB2-Fab-TCO, M: molecular marker); (**D**) UPLC profile of anti-ErbB3-Fab-IEDDA-anti-ErbB2-bsFab conjugate after purification via SEC. Light grey Fab: anti-ErbB3-Fab-MeTz, dark grey Fab: anti-ErbB2-Fab-TCO. Reaction conditions: 30 µM anti-ErbB3-Fab-MeTz, 60 µM anti-ErbB2-Fab-TCO in PBS, (**A**): 30–50% acetonitrile/ddH_2_O in 10 min, (**D**): 10–80% acetonitrile/ddH_2_O in 10 min.

The subsequent SPAAC- and IEDDA-based click reactions were performed according to established protocols (Section 3.5). The course and analysis of the reactions are shown in Figure 4 using the IEDDA reaction as an example. As for the SPAAC reaction (Figure S1), a quantitative product yield could also be obtained for the IEDDA coupling at a stoichiometry of 1:2 of the starting substrates. The only differences were in the reaction times, which ranged from several minutes to a few hours. In fact, the IEDDA-based click reaction was completed within about 30 min (Figure 4A). The SPAAC reaction, on the other hand, took about 4 h to reach complete conversion (Figure S1B,C). Regardless of the individual reaction time, in all cases, the formation of the desired conjugates (Figure 4A,B and Figure S1B,C) could be detected after only a few seconds (Figure 4C and Figure S1D). The remaining bands after complete conversion at 40–55 kDa in the SDS-PAGE of Figure 4C (and Figure S1D, respectively) corresponded to unseparated, unmodified Fab species from the enzymatic reaction. These and the excess click component were finally separated by SEC in a one-step purification, yielding a final bispecific product of high purity and homogeneity (Figure 4D and Figure S1E).

#### 2.2.2. Enzymatic Synthesis of *C*- to *C*-Linked Anti-ErbB2-Anti-ErbB3-bsFab

**Figure 5:**
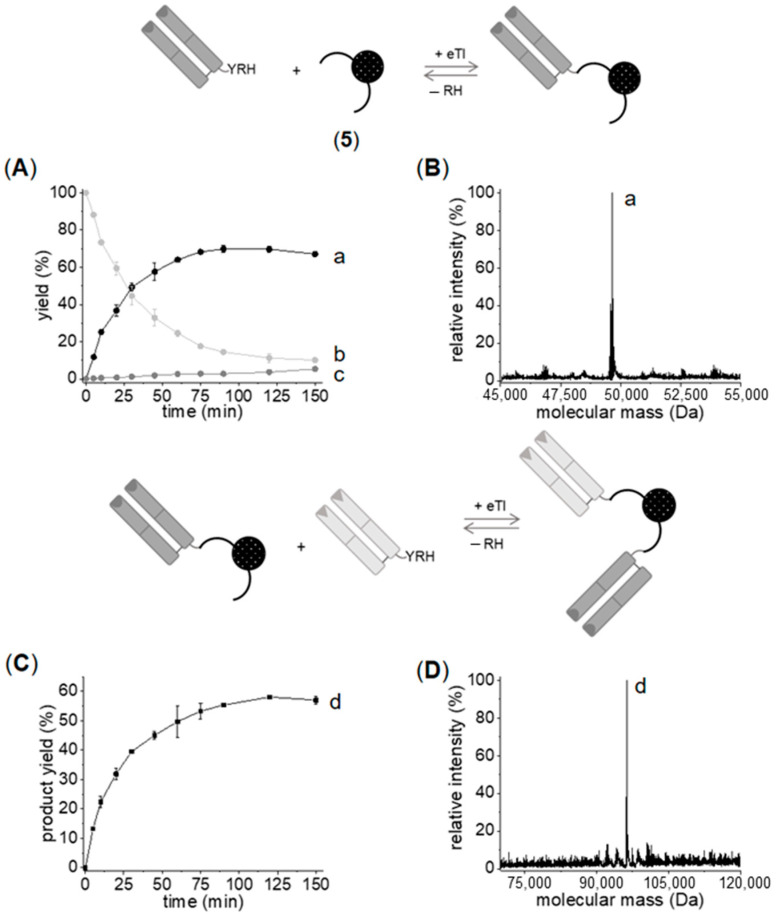
Results of the enzymatic *C*- to *C*-terminal coupling of anti-ErbB2-Fab-YRH and anti-ErbB3-Fab-YRH via linker **5**. (**A**) Reaction kinetics: the single modified product (black line) was mainly formed while only traces of the homodimeric product (dark grey line) were generated. a: anti-ErbB2-Fab-linker **5**, b: anti-ErbB2-Fab-YRH, c: homodimeric anti-ErbB2-linker **5**-anti-ErbB2-Fab; (**B**) MS analysis of the product purified by HIC, a: anti-ErbB2-Fab-linker **5** M_calcd_: 49,538 Da, M_found_: 49,539 Da; (**C**) Reaction course of anti-ErbB2-linker **5**-anti-ErbB3-bsFab synthesis with yields of about 60% analyzed by HIC, d: anti-ErbB2-linker **5**-anti-ErbB3-bsFab; (**D**) MS analysis of the purified bsFab, anti-ErbB2-linker **5**-anti-ErbB3-bsFab M_calcd_: 96,244 Da, M_found_: 96,245 Da. Reaction conditions: (**A**): 100 µM anti-ErbB2-Fab-YRH, 500 µM linker **5**, 10 µM eTl, 100 µM ZnCl_2_; (**C**): 20 µM anti-ErbB2-Fab-linker **5**, 100 µM anti-ErbB3-Fab-YRH, 2.5 µM eTl, 50 µM ZnCl_2_; (**A**,**C**): 100 mM HEPES/NaOH, pH 7.8, 100 mM NaCl, 10 mM CaCl_2_.

The eTl-catalyzed *C*- to *C*-linkage of two Fabs necessarily requires a special linker structure equipped with two nucleophilic recognition sequences for the biocatalyst (RH-motifs). Linker **5** obviously fulfilled this requirement. Furthermore, two Fab substrates were required, each carrying the recognition sequence YRH for eTl at the *C*-terminus. The reaction setting consisting of linker **5** and anti-ErbB2-Fab-YRH and anti-ErbB3-Fab-YRH fulfilled both requirements but bore the general risk that, in addition to the bispecific product, the respective homodimers are simultaneously formed from anti-ErbB2-Fab or anti-ErbB3-Fab. A sequential reaction mode, starting first with the coupling of anti-ErbB2-Fab-YRH with the linker and second, the coupling of anti-ErbB3-Fab-YRH to the resulting intermediate could minimize this risk, especially if the nucleophilic component (linker **5**) was used in excess. Since the latter was the standard case in all previously performed eTl reactions, the reaction conditions were maintained. The results of both reactions are shown in Figure 5.

As it can be seen in Figure 5A, for the first reaction of the two-step procedure, the coupling of anti-ErbB2-Fab-YRH with linker **5**, a product yield of higher 70% was obtained after approximately 75 min of reaction time. This corresponded to the results obtained with linkers **1** to **4**. Interestingly, only about 3% of the homodimeric anti-ErbB2-linker **5**-anti-ErbB2-Fab was formed (Figure 5A). Apparently, the 5-fold excess of the nucleophilic linker used was already sufficient to almost completely prevent unwanted homodimerization. After the monomeric anti-ErbB2-Fab-linker **5** product was purified by means of hydrophobic interaction chromatography (HIC) (Figure 5B), the second eTl-catalyzed coupling step was carried out by adding anti-ErbB3-Fab-YRH under again analogous reaction conditions. The result of this second enzymatic reaction is shown in Figure 5C,D. Corresponding to the course of the reaction shown in Figure 5C, a product yield of approx. 60% of the desired anti-ErbB2-Fab-linker **5**-anti-ErbB3-bsFab could be obtained in a reaction time of approx. 120 min. This yield corresponded to the *C*- to *N*-terminal coupling of the two Fabs by eTl (Section 2.1). Finally, the bispecific product was isolated and purified by SEC (Figure 5D) and subsequently used for functional studies (Section 2.3). Noticeably, with this method, it was rather impossible to control which Fab was attached to which site of the linker, which is, however, without relevance if the linker structure is symmetric. In cases where control is essential, two orthogonal Trypsiligase variants could be used in a one-step procedure to address this problem [25].

### 2.3. Analysis of In Vitro Functionality of the Generated bsFabs

#### 2.3.1. Surface Plasmon Resonance (SPR)-Based Activity Assay

The in vitro function of all synthesized and purified anti-ErbB2-anti-ErbB3-bsFabs was first analyzed regarding their epitope-binding behavior. For this purpose, the dissociation constants (K_D_) were investigated using an SPR-based activity assay compared to the single ErbB2- and ErbB3-Fabs. The K_D_ values obtained were determined sequentially by immobilizing the ectodomains of the receptors ErbB2 and ErbB3 on separate sensor chip surfaces and are summarized in Table 1. The complete SPR sensorgrams as well as the values for k_on_ and k_off_ are shown in Figure S6 and Figure S7. First, for the single anti-ErbB2- and anti-ErbB3-Fabs, it was noted that neither showed any cross-reactivity in their antigen-binding properties (Table 1). Second, it is clear from Table 1 that the additional *N*-terminal RH-motif in the anti-ErbB2-Fab did not affect the dissociation constant. As a consequence, the K_D_ values were in a narrow range and correspond to those described in the literature [23,33]. Third, the results further showed that all bsFabs synthesized retained their binding functionality. The determined dissociation constants were comparable to those of the individual single Fabs. Fourth, even the binding behavior of the inner domain of the *C*- to *N*-terminal linked bsFab format (anti-ErbB2-domain) did not appear to be affected by the outer anti-ErbB3-domain. Thus, it can be concluded that the type of linkage in which the two individual ErbB2 and ErbB3 Fabs are fused (at least as it is in the synthesized formats), has no significant influence on the binding properties to the antigens.

#### 2.3.2. Receptor Internalization Assay

The internalization studies were conducted with three breast cancer cell lines. These cell lines differ in the number of ErbB2 and ErbB3 receptors expressed on their cell surfaces. SKBR-3 cells show high levels of both receptors, while HCC-1954 cells are known for high ErbB2 and low ErbB3 receptor expression. In contrast, the reverse holds true for MCF-7 cells. They are characterized by low ErbB2 and high ErbB3 receptor levels [35]. It was expected that the bsFab formats, in addition to binding the respective specific antigens, would also exhibit synergistic binding in the presence of both antigens on the cell surface.

**Figure 6:**
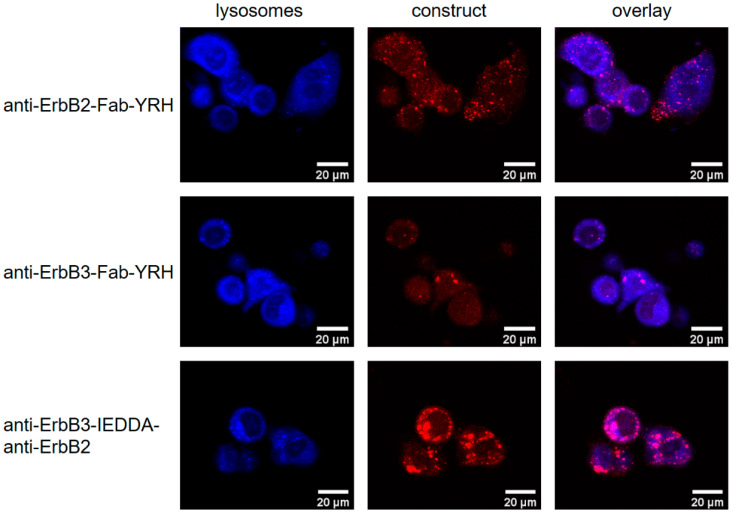
Internalization of anti-ErbB3-IEDDA-anti-ErbB2-bsFab compared to the single anti-ErbB2- and anti-ErbB3-Fab by lysosome-stained SKBR-3 cells after 24 h. Lysosomes were stained with LysoBriteBlue, single and bispecific Fabs were modified with AlexaFluor568 NHS ester. IEDDA: inverse electron-demand Diels–Alder reaction.

First, all single and bispecific Fabs to be measured were modified non-specifically with an AlexaFluor568 NHS ester for the internalization studies. The resulting dye loading varied from 2 to 11 depending on the protein used. SDS-PAGE and UV/Vis absorption spectroscopic analyses showed no evidence of aggregate formation, especially at higher dye loadings (Figure S8). In addition, a lysosomal dye was used to follow the path of the proteins in the cells. The progression of internalization is shown for anti-ErbB3-IEDDA-anti-ErbB2-bsFab and SKBR-3 cells as an example of all bsFabs in Figure 6. In addition, the course of internalization of every single anti-ErbB2- and anti-ErbB3-Fab is shown. The complete data sets for all bsFabs and cell lines can be found in Figures S2–S5 in the Supplementary Materials. In general, little fluorescence intensities for all Fabs/bsFabs were observed for the MCF-7 cell line (Figure S3). In contrast, for SKBR-3 cells, significant fluorescence signals of all added proteins corresponding to that of the lysosomes were found, indicating internalization (Figure 6). Remarkably, the fluorescence signals of the bsFabs in the case of the SKBR-3 cells were much stronger than that of the single Fabs alone. The fluorescence intensity of the single anti-ErbB3-Fab was worse compared to that of the single anti-ErbB2 counterpart. A more in-depth quantification of these tendencies based on the performed fluorescence microscopic analyses was, however, not possible with certainty. Additional flow cytometric studies are recommended for this purpose, but these were beyond the focus of this work.

**Figure 7:**
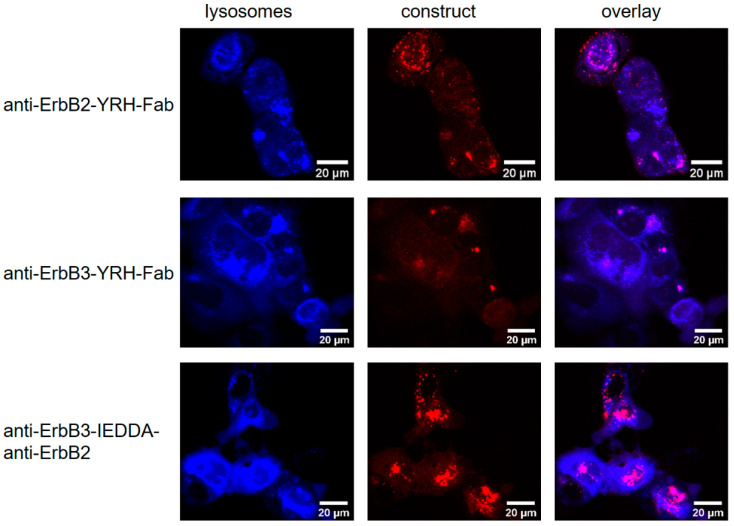
Internalization of anti-ErbB3-IEDDA-anti-ErbB2-bsFab compared to the single anti-ErbB2- and anti-ErbB3-Fab by lysosome-stained HCC-1954 cells after 24 h. Lysosomes were stained with LysoBriteBlue, single and bispecific Fabs were modified with AlexaFluor568 NHS ester. IEDDA: inverse electron-demand Diels–Alder reaction.

**Figure 8:**
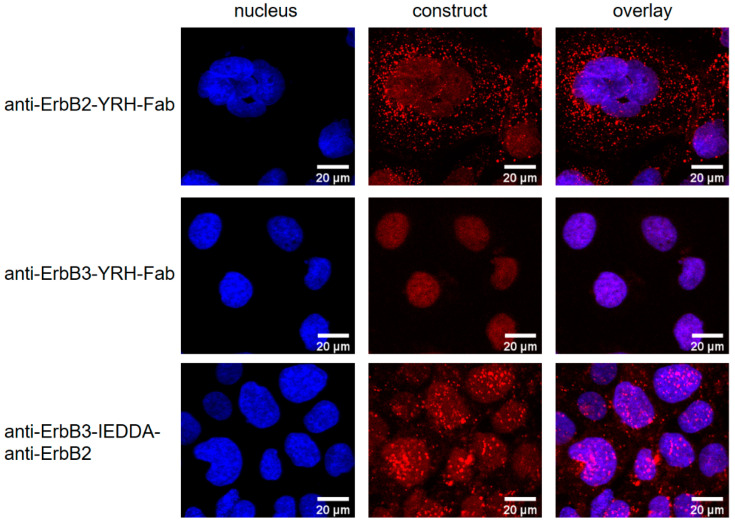
Internalization of anti-ErbB3-IEDDA-anti-ErbB2-bsFab compared to the single anti-ErbB2- and anti-ErbB3-Fab by cell nucleus-stained HCC-1954 cells after 24 h. Substantial portions of the bsFabs could be found in the cell nucleus. Cell nuclei were stained with HOECHST33342, single and bispecific Fabs were modified with AlexaFluor568 NHS ester. IEDDA: inverse electron-demand Diels–Alder reaction.

In the case of HCC-1954 cells, a similar trend to SKBR-3 cells was observed (Figure 7). While the fluorescence spots for the single Fabs were still congruent with the lysosomes, this was only partially the case for the bsFabs. A plausible explanation could be that the bsFab-receptor complexes are translocated into the nucleus via the non-canonical pathway [36,37,38]. To test this hypothesis initially, we performed the same experiment with nuclear instead of lysosome staining (Figure 8). While the fluorescence signal of the single anti-ErbB2-Fab-YRH was mainly found distant from that of the nucleus, the signals of all bsFabs could be found both distant and superimposed on the nucleus. This finding may indicate a different distribution of mono- and bispecifics in HCC-1954 cells. Whether the bsFabs can actually be found on or even in the cell nucleus cannot be concluded with certainty from this finding.

## 3. Materials and Methods

### 3.1. Chemicals and Peptide Synthesis

All reagents were purchased in the highest quality available at Sigma–Aldrich (St. Louis, MO, USA). Peptides were synthesized following standard procedures using Fmoc/tBu strategy [24,39,40]. Amino acids and reagents for peptide synthesis were purchased at IRIS Biotech (Marktredwitz, Germany). Click reagents were purchased at Jena Bioscience (Jena, Germany) and IRIS Biotech (Marktredwitz, Germany).

H-RHAC-OH peptide and maleimide click reagents were dissolved in a phosphate buffer (100 mM NaH_2_PO_4_/NaOH, 150 mM NaCl, pH 7.5) with a maximum of 10% (*v*/*v*) DMF. Reactants were mixed in a ratio of 1:1.1 (peptide to click component) and were incubated for 2 h at room temperature. Purification was done by preparative HPLC (waters system (Milford, MA, USA), XSelect^®^ Peptide CSH^TM^ C18) with a linear gradient of 5–95% acetonitrile/ddH_2_O in 40 min. The identity of the synthesis products was verified by electrospray ionization mass spectrometry (waters Micromass^®^ ZQ™ (Milford, MA, USA)) and the purity was determined by UPLC (5–95% acetonitrile/ddH_2_O in 4 min at 220 nm; waters system (Milford, MA, USA), ACQUITY BEH 130, C18, 1.7 µm, 2.1 × 100 mm).

Analysis: H-RHAC(Mal-PEG_3_-TCO)-OH: LC-MS (ESI^+^) m/z: 1008.5 [M]^+^, calcd. for [C_44_H_72_N_12_O_13_S]^+^ m/z: 1009.3, purity: 94.1%; H-RHAC(Mal-PEG_4_-MeTz)-OH: LC-MS (ESI^+^) m/z: 999.8 [M]^+^, calcd. for [C_42_H_61_N_15_O_12_S]^+^ m/z: 999.4, purity: 97.7%; H-RHAC(Mal-PEG_4_-DBCO)-OH: LC-MS (ESI^+^) m/z: 1160.8 [M]^+^, calcd. for [C_54_H_73_N_13_O_14_S]^+^ m/z: 1159.5, purity: 97.1%; H-RHAK(PAA)-OH: LC-MS (ESI^+^) m/z: 636.1 [M]^+^, calcd. for [C_26_H_45_N_13_O_6_]^+^ m/z: 635.4, purity: 91.5%; H-RHAGGK(H-RHAGG)GGWGGK(N_3_)-OH: LC-MS (ESI^+^) m/z: 1671.5 [M]^+^, calcd. for [C_69_H_106_N_32_O_18_]^+^ m/z: 1670.8, purity: 95.3%.

### 3.2. Production of eTl

The eTl encoding plasmid (pPICZaA) was linearized by *Sac*I-HF (NEB (Ipswich, MA, USA) and purified by agarose gel electrophoresis (NucleoSpin Gel and PCR Clean-up-Kit (Macherey and Nagel (Düren, Germany)).

*Pichia pastoris* X-33 cells were electroporated according to the protocol described in [41]. The selection of positive clones was performed by Zeocin (200 µg/mL). Cells were cultivated for 24 h, 30 °C in YNB-medium (13.4 g/L YNB (w/o aa), 50 mM MES/NaOH; pH 6.0, 10 mM CaCl_2_ with 20% (*m*/*v*) glucose and then pelleted and transferred in YNB-medium with 1% (*v*/*v*) methanol. After 3 days, the supernatant was harvested and diluted in IEX buffer (1:1) (20 mM Na-acetate/acetic acid; pH 4.0). After loading on a cation exchange column (HiPrep SP FF, Cytiva (Chalfont St Giles, UK)), the protein was eluted with 100 mM HEPES/NaOH; pH 7.8, 200 mM NaCl, 10 mM CaCl_2_. Final purification was done by SEC (S75 pg, 16/600, Cytiva (Chalfont St Giles, UK)) in HEPES-buffer (100 mM HEPES/NaOH, pH 7.8, 100 mM NaCl, 10 mM CaCl_2_).

Analysis: Calculated molecular mass: M_calcd_: 23,758 Da, M_found_: 23,759 Da.

### 3.3. Production of Fabs

The anti-ErbB3-Fab was designed according to the protocol described in [33]. Anti-ErbB3-Fab-YRH and anti-ErbB2-Fab-YRH have a prolonged *C*-terminal protein sequence at the heavy chain with the sequence: PGGYRHAAGEQKLISEEDL [25]. The *N*-terminally extended anti-ErbB2-RH-Fab was prolonged at the heavy chain with the amino acid sequence RHA, while the RH-motif represents the enzyme recognition tag, an A serves as an additional spacer amino acid. The pASK-anti-ErbB2-Fab plasmid and the pET-anti-ErbB3-Fab plasmid were transformed into *E. coli* BL21 (DE3) cells. Cultures were grown at 37 °C with either 100 µg/mL ampicillin or 50 µg/mL kanamycin until OD_600nm_ of 0.8 was reached. Induction was done by adding 0.1 µg/mL anhydrotetracycline and 0.5 mM IPTG, respectively, for 4 h, 30 °C. Cell pellets were resuspended in phosphate buffer (50 mM NaH_2_PO_4_; pH 7.0) and lysed by sonification (amplitude 30%). The Fabs were purified via Protein G or Protein A affinity chromatography (HiTrap Protein G or A HP, Cytiva (Chalfont St Giles, UK)) and SEC (S75 pg, 16/600) in HEPES-buffer (100 mM HEPES/NaOH, pH 7.8, 100 mM NaCl, 10 mM CaCl_2_).

Analysis: Calculated molecular masses for Fabs: anti-ErbB3-Fab-YRH M_calcd_: 48,401 Da, M_found_: 48,402 Da; anti-ErbB2-Fab-YRH M_calcd_: 49,548 Da, M_found_: 49,549 Da; anti-ErbB2-RH-Fab M_calcd_: 48,099 Da, M_found_: 48,100 Da.

### 3.4. C-Terminal Modification of Fabs by eTl

The following standard reaction conditions were used for eTl catalyzed transamidation reactions: 100 μM Fab, 100 μM ZnCl_2_ in HEPES buffer (100 mM HEPES/NaOH, pH 7.8, 100 mM NaCl, 10 mM CaCl_2_) in a reaction volume of 100–500 μL. The nucleophile was freshly dissolved in buffer and added to Fab in 5-fold molar excess. The reaction was initiated by adding 10 µM of eTl. Incubation was performed at 30 °C, 550 rpm for 150 min. Reaction kinetics were examined by HIC according to [24] with a linear gradient from 95% equilibration buffer (1.5 M ammonium sulfate, 0.05 HEPES/NaOH; pH 7.5) to 0% in 15 min, SDS-PAGE [23,24,42] (molecular marker: PageRuler Plus pre-stained protein ladder (ThermoFisher Scientific (Waltham, MA, USA)) and LC-MS analysis [24].

### 3.5. Chemo–Enzymatic Production of bsFabs

A standard reaction mixture was prepared: 100 μM Fab, 500 µM linker **1**–**4**, 10 µM eTl, 100 μM ZnCl_2_ in HEPES buffer (100 mM HEPES/NaOH, pH 7.8, 100 mM NaCl, 10 mM CaCl_2_). When reaching the product maximum, the mixture was diluted in phosphate buffer (50 mM NaH_2_PO_4_; pH 7.0) (1:5). Anti-ErbB2- and anti-ErbB3-Fab mixtures were loaded onto a Protein G and A Spin Column, respectively (NAb Protein G or A Spin Columns, ThermoFisher Scientific (Waltham, MA, USA)). Purification was performed by 5 washing steps with phosphate buffer (50 mM NaH_2_PO_4_; pH 7.0) and 4 elution steps (100 mM glycine/HCl; pH 2.7). The elution fractions were pooled, and the buffer was exchanged by cross filtration to phosphate-buffered saline (PBS, 137 mM NaCl, 2.7 mM KCl, 10 mM Na_2_HPO_4_, 1.8 mM KH_2_PO_4_; pH 7.4). Click anchor modified Fabs were mixed together in a ratio of 1:2 according to the manufacturer’s instructions (Jena Bioscience (Jena, Germany)) for the respective click reaction type and monitored by 10% SDS-PAGE, UPLC (waters system (Milford, MA, USA), Aeris^TM^ 3.6 µm WIDEPORE XB-C18 column, Phenomenex (Torrance, CA, USA)) and LC-MS. Purification of the bsFabs was done by SEC (S200 pg, 16/600, Cytiva (Chalfont St Giles, UK)) in PBS.

### 3.6. Enzymatic Production of C- to C- and C- to N-Terminal bsFabs

For the *C*- to *C*-terminal Fab couplings, a standard reaction mixture was prepared: 100 μM anti-ErbB2-Fab-YRH, 500 µM linker **5**, 10 µM eTl, 100 μM ZnCl_2_ in HEPES buffer (100 mM HEPES/NaOH, pH 7.8, 100 mM NaCl, 10 mM CaCl_2_). When reaching the maximum product yield, the mixture was purified by HIC. The single modified product was concentrated, buffer exchanged by cross filtration to HEPES buffer (100 mM HEPES/NaOH, pH 7.8, 100 mM NaCl, 10 mM CaCl_2_) and used for the following modification step. A standard mixture was prepared to generate either the *C*- to *C*- or *C*- to *N*-terminal bsFab (100 µM anti-ErbB3-Fab-YRH, 20 µM modified product or anti-ErbB2-RH-Fab, 2.5 µM eTl, 50 µM ZnCl_2_ in HEPES buffer (100 mM HEPES/NaOH, pH 7.8, 100 mM NaCl, 10 mM CaCl_2_)). Upon reaching the maximum product yield, the reaction mixture was diluted in 100 mM glycine/HCl; pH 2.7 and incubated for 10 min. Purification was done by SEC (S200 pg, 16/600) in PBS.

### 3.7. SPR-Based Activity Assay

SPR spectroscopy was performed on a BIAcore X instrument (BIAcore (Uppsala, Sweden)). The biotinylated ErbB2 or ErbB3 ectodomains (BioCat, (Heidelberg, Germany)) were immobilized, each on a separate SAHC 200 M sensor chip (XanTec bioanalytics (Düsseldorf, Germany)), resulting in a surface density of approximately 1600 RU. The Fabs or bsFabs were applied in a dilution series using PBS as running buffer. Complex formation was observed at a continuous flow rate of 30 μL/min for 120 s. Kinetic parameters were determined by fitting the data to the 1:1 Langmuir binding model with the BIAevaluation software (BIAcore (Uppsala, Sweden)). After each injection, the surface was regenerated by injecting 10 µL 10 mM NaOH, 1 M NaCl.

### 3.8. Receptor Internalization Assay

Fluorescence labeling of the proteins was performed according to the AlexaFluor568 carboxylic acid NHS ester manufacturer’s instructions (ThermoFisher Scientific (Waltham, MA, USA)). SKBR-3-Luc (JCRB1627.1), MCF-7-Luc (JCRB1372), and HCC-1954-Luc (JCRB1476) were purchased from JCRB (Tokyo, Japan). For internalization 5000 cells per well were seeded in a µ-slide 8-well chamber coverslip (ibidi (Gräfeling, Germany)) and incubated for 48 h at 37 °C, 5% CO_2_ (RPMI1640, 10% (*v*/*v*) fetal bovine serum (FBS), ThermoFisher Scientific (Waltham, MA, USA)). The medium was replaced by FluoroBrite DMEM medium with 10% (*v*/*v*) FBS (ThermoFisher Scientific (Waltham, MA, USA)) and 50 nM labeled Fab or bsFab was added and incubated for 24 h. Before starting microscopy, cells were washed with FluoroBrite DMEM medium without FBS. Cells were mixed with equivalent amounts of FluoroBrite DMEM medium and life cell staining buffer (supplemented with either LysoBriteBlue (1:500), AATBioquest (Sunnyvale, CA, USA), or HOECHST33342 (1:2000), ThermoFisher Scientific (Waltham, MA, USA)) and incubated prior to microscopy for 0.5–2 h and 10 min, respectively (Eclipse TE2000-E, CFI Plan Fluor 40x oil lens, Nikon (Tokyo, Japan)). Lysosome and nucleus staining were excited at 405 nm (filter 450/35), fluorescent proteins at 568 nm (filter 650LP).

## 4. Conclusions

Currently, more than one hundred formats of bsAbs have been described in the scientific literature. Not infrequently, the format determines the therapeutic efficacy. In this study, we presented an approach that can generate numerous amounts of such formats with only a single enzyme. By combining this with click chemistry techniques, a modular synthesis kit was created that allowed rapid and flexible shuffling of the individual antigen-binding domains in different but well-defined arrangements. The formats generated in this way can then be used to screen potentially suitable candidates for the respective application. The heterologous expression of each individual format or the use of different coupling procedures with individual reaction conditions, including the need for two distinct enzymes, as is currently the case, is not necessary. We were able to show that both the purely enzymatic synthesis and the chemo–enzymatic reactions enable high product yields with only short reaction times. The products were homogeneous and showed a uniform architecture. The formation of multimeric synthesis products, as found with the use of transglutaminase [18], could not be observed with eTl. We were also able to initially demonstrate the biological function of all bispecific constructs. As expected, we could show that the synthesized and fluorescence-labeled anti-ErbB2-anti-ErbB3-bsFabs exhibited improved fluorescence intensities in mammalian breast cancer cell lines compared with the single Fabs alone. Our results suggest that the bispecific formats produced might follow a partially different endocytotic pathway after internalization in certain cell lines than the individual Fabs from which they were constructed. In particular, the findings with labeled bsFabs, which in the case of HCC-1954 cells lead to additional fluorescence signals overlapping with those of the nuclei, give rise to further studies. The background for this is the clinical finding that the diagnosis of EGF receptors localized in the cell nucleus is associated with poor patient prognosis in the case of severe cancer progression [43,44]. On the basis of these findings, it would be very promising to investigate whether the transport of DNA-damaging toxins in the direction of the cell nucleus mediated by the bsFabs may lead to additional effects on the cancer cell compared to the monospecific Fabs. With the trifunctional linker already used in this study, this would be possible without any problems from a synthetic point of view and with high flexibility in terms of the active substance. Studies in this direction are presently in progress.

## Figures and Tables

**Table 1:** Comparison of dissociation constants of single and bispecific ErbB2- and ErbB3-Fabs determined by SPR. Biotinylated ErbB2 or ErbB3 ectodomains were immobilized separately on streptavidin sensor chips; no binding was detected. SPAAC: strain-promoted alkyne-azide cycloaddition, IEDDA: inverse electron-demand Diels–Alder reaction.

	ErbB3-ECD K_D_ (pM)	ErbB2-ECD K_D_ (pM)
anti-ErbB3-Fab-YRH	99 ± 22	-
anti-ErbB2-Fab-YRH	-	139 ± 31
anti-ErbB2-RH-Fab	-	137 ± 43
anti-ErbB2-SPAAC-anti-ErbB3	73 ± 21	130 ± 28
anti-ErbB3-IEDDA-anti-ErbB2	63 ± 19	113 ± 20
anti-ErbB3-YRH-anti-ErbB2	95 ± 45	138 ± 46
anti-ErbB2-linker **5**-anti-ErbB3	97 ± 33	108 ± 20

## Supplementary Materials

The following material is available online at https://www.mdpi.com/article/10.3390/ijms23063144/s1.
