# Supplementary material for: Synthesis of Multiple Bispecific Antibody Formats with Only One Single Enzyme Based on Enhanced Trypsiligase
Source: Int J Mol Sci. 2022 Mar 15;23(6):3144. doi: 10.3390/ijms23063144 (PMC8952323; doi:10.3390/ijms23063144)
Supplement: Supplementary file 1 [file ijms-23-03144-s001.zip › ijms-1513134-supplementary.pdf]

Supplementary Materials for

Synthesis of multiple bispecific antibody formats with only one single enzyme based on enhanced Trypsiligase

Johanna Voigt, Christoph Meyer and Frank Bordusa

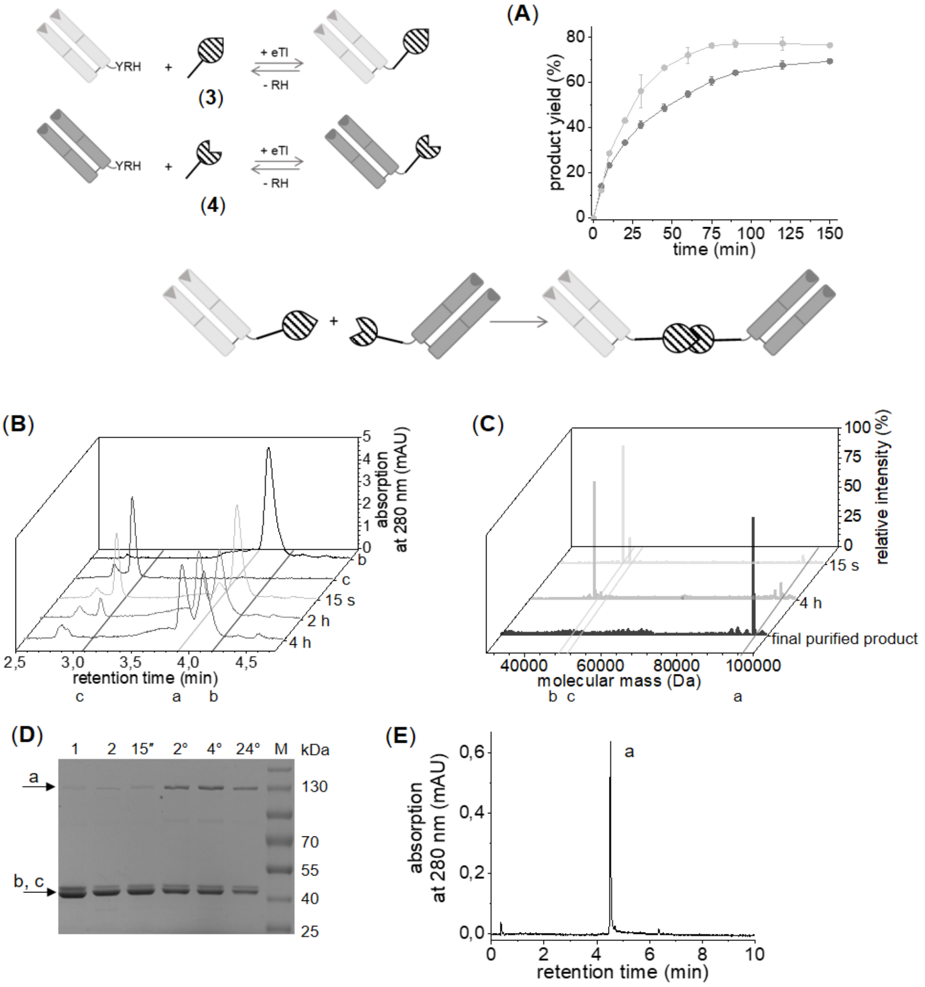

Figure S1: Coupling of anti-ErbB2-Fab-YRH and anti-ErbB3-Fab-YRH to click linker **3** and **4** via eTl-catalysis followed by click coupling of the resulting intermediate products anti-ErbB2-Fab-DBCO and anti-ErbB3-Fab-PAA forming the final anti-ErbB3-SPAAC-anti-ErbB2-bsFab. (A) Reaction kinetics with maximum product yields higher 70%, light grey line: anti-ErbB2-Fab-DBCO, grey line: anti-ErbB3-Fab-PAA; (B and C) Analysis of click reaction of the click anchor modified intermediate Fabs to the final bsFab. The click reaction is completed within 4 h analysed by (B) UPLC-analysis and (C) MS analysis, bispecific product purified by SEC, a: anti-ErbB2-SPAAC-anti-ErbB3-bsFab M_{calcd}: 96352 Da, M_{found}: 96353 Da, b: anti-ErbB3-Fab-PAA M_{calcd}: 47.340 Da, M_{found}: 47.341 Da, c: anti-ErbB2-Fab-DBCO M_{calcd}: 49.012 Da, M_{found}: 49.012 Da; (D) Time-resolved SDS-PAGE analysis of the click reaction (1: anti-ErbB2-Fab-DBCO, 2: anti-ErbB3-Fab-PAA, M: molecular marker); (E) UPLC profile of anti-ErbB3-SPAAC-anti-ErbB2-bsFab conjugate after purification via SEC. Light grey Fab: anti-ErbB3-Fab, dark grey Fab: anti-ErbB2-Fab; eTl: enhanced Trypsiligase.

Reaction conditions: A: 100 µM anti-ErbB2-Fab-YRH /anti-ErbB3-Fab-YRH , 500 µM linker **3** or linker **4**, 10 µM eTl, 100 µM $ZnCl_2$, 5% (v/v) DMSO, 100 mM HEPES/NaOH, pH 7.8, 100 mM NaCl, 10 mM $CaCl_2$; B-D: 30 µM anti-ErbB2-Fab-DBCO, 60 µM anti-ErbB3-Fab-PAA in PBS, B: 30 - 50% acetonitrile/ddH_2O in 10 min, E: 10 - 80% acetonitrile/ddH_2O in 10 min

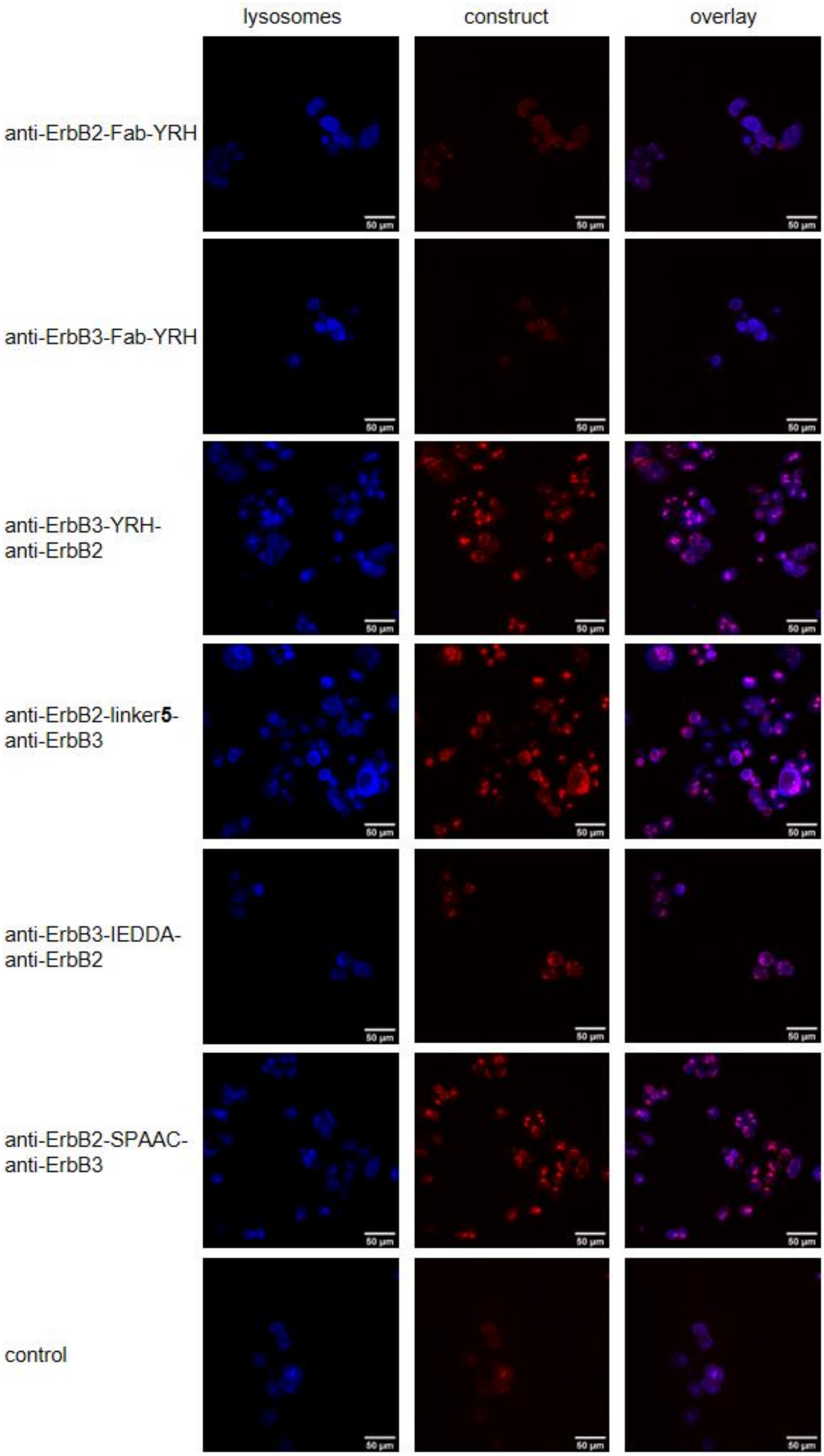

Figure S2: Internalisation of the anti-ErbB3-anti-ErbB2-bsFab formats compared to the single anti-ErbB2- and anti-ErbB3-Fab in SKBR-3 cells after 24 h. Lysosomes are stained with LysoBriteBlue, single and bispecific Fabs are modified with AlexaFluor568 NHS ester. PBS was used as control instead of the Fabs or bsFabs. SPAAC: strain-promoted alkyne-azide cycloaddition, IEDDA: inverse electron-demand Diels-Alder reaction

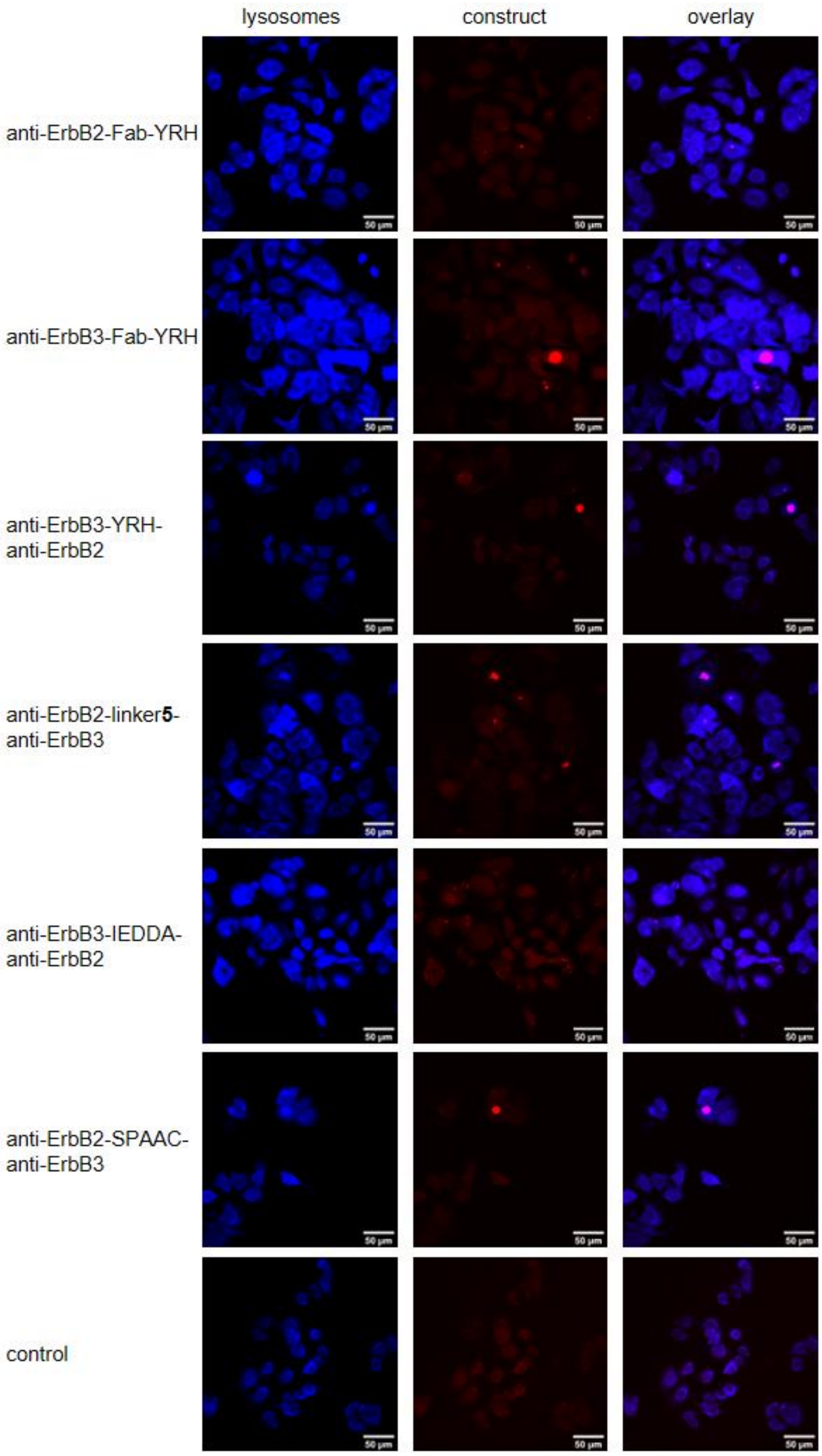

Figure S3: Internalisation of the anti-ErbB3-anti-ErbB2-bsFab formats compared to the single anti-ErbB2- and anti-ErbB3-Fab in MCF-7 cells after 24 h. Lysosomes are stained with LysoBriteBlue, single and bispecific Fabs are modified with AlexaFluor568 NHS ester. PBS was used as control instead of the Fabs or bsFabs. SPAAC: strain-promoted alkyne-azide cycloaddition, IEDDA: inverse electron-demand Diels-Alder reaction

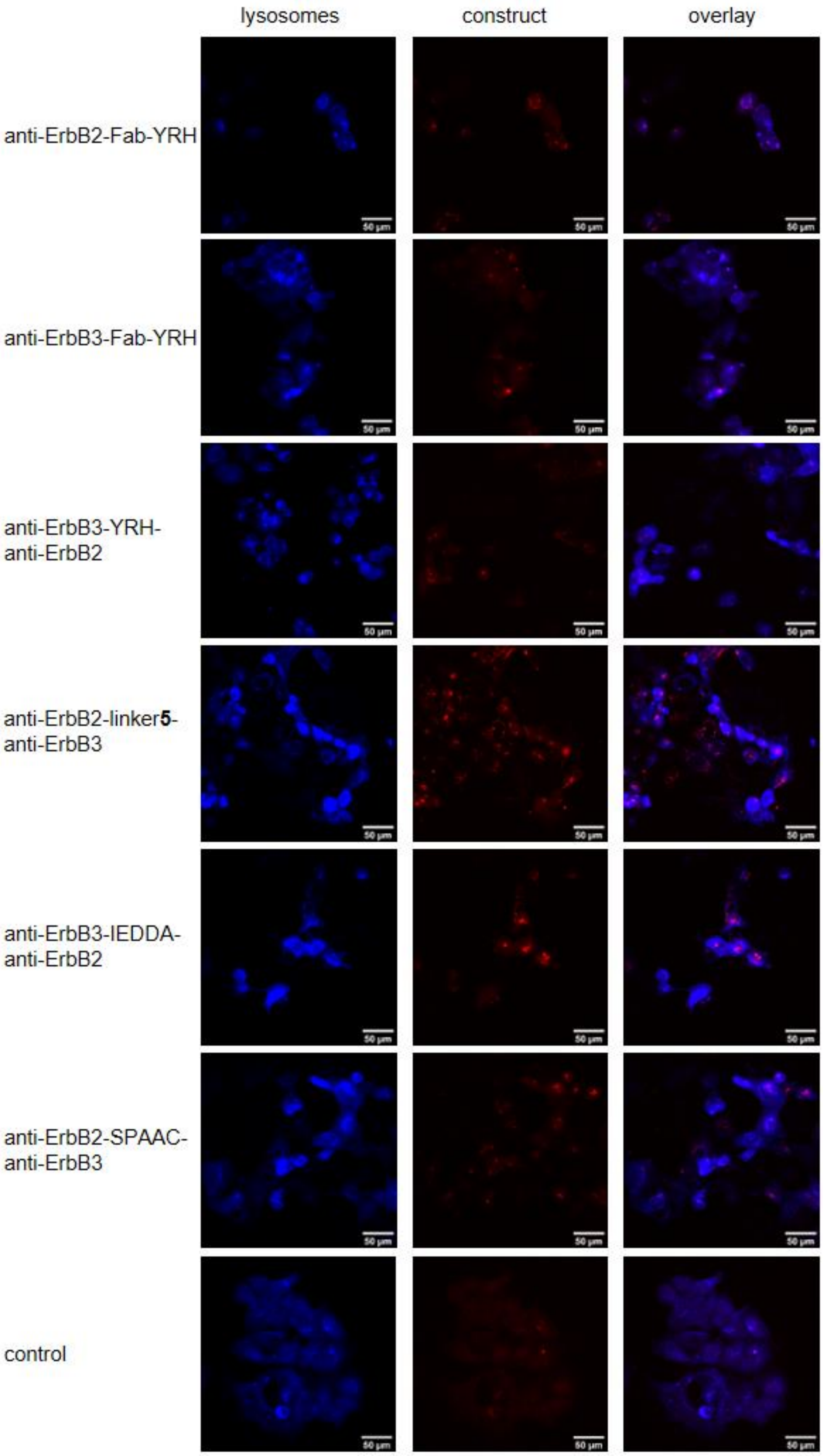

Figure S4: Internalisation of the anti-ErbB3-anti-ErbB2-bsFab formats compared to the single anti-ErbB2- and anti-ErbB3-Fab in HCC-1954 cells after 24 h. Lysosomes are stained with LysoBriteBlue, single and bispecific Fabs are modified with AlexaFluor568 NHS ester. PBS was used as control instead of the Fabs or bsFabs. SPAAC: strain-promoted alkyne-azide cycloaddition, IEDDA: inverse electron-demand Diels-Alder reaction

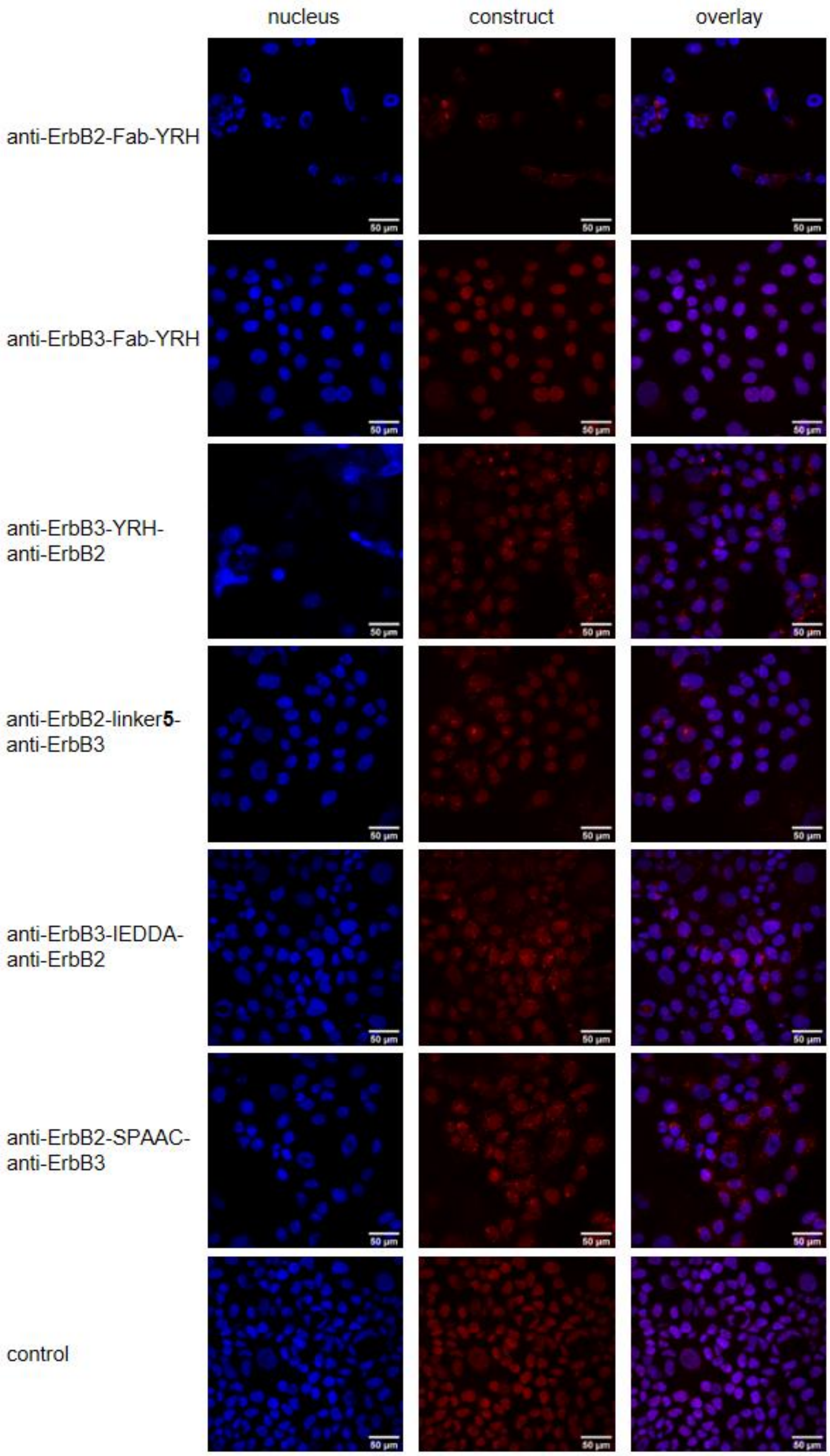

Figure S5: Internalisation of the anti-ErbB3-anti-ErbB2-bsFab formats compared to the single anti-ErbB2- and anti-ErbB3-Fab by cell nucleus-stained HCC-1954 cells after 24 h. Substantial portions of the bsFabs can be found in the cell nucleus. Cell nuclei are stained with HOECHST33342, single and bispecific Fabs are modified with AlexaFluor568 NHS ester. PBS was used as control instead of the Fabs or bsFabs. SPAAC: strain-promoted alkyne-azide cycloaddition, IEDDA: inverse electron-demand Diels-Alder reaction

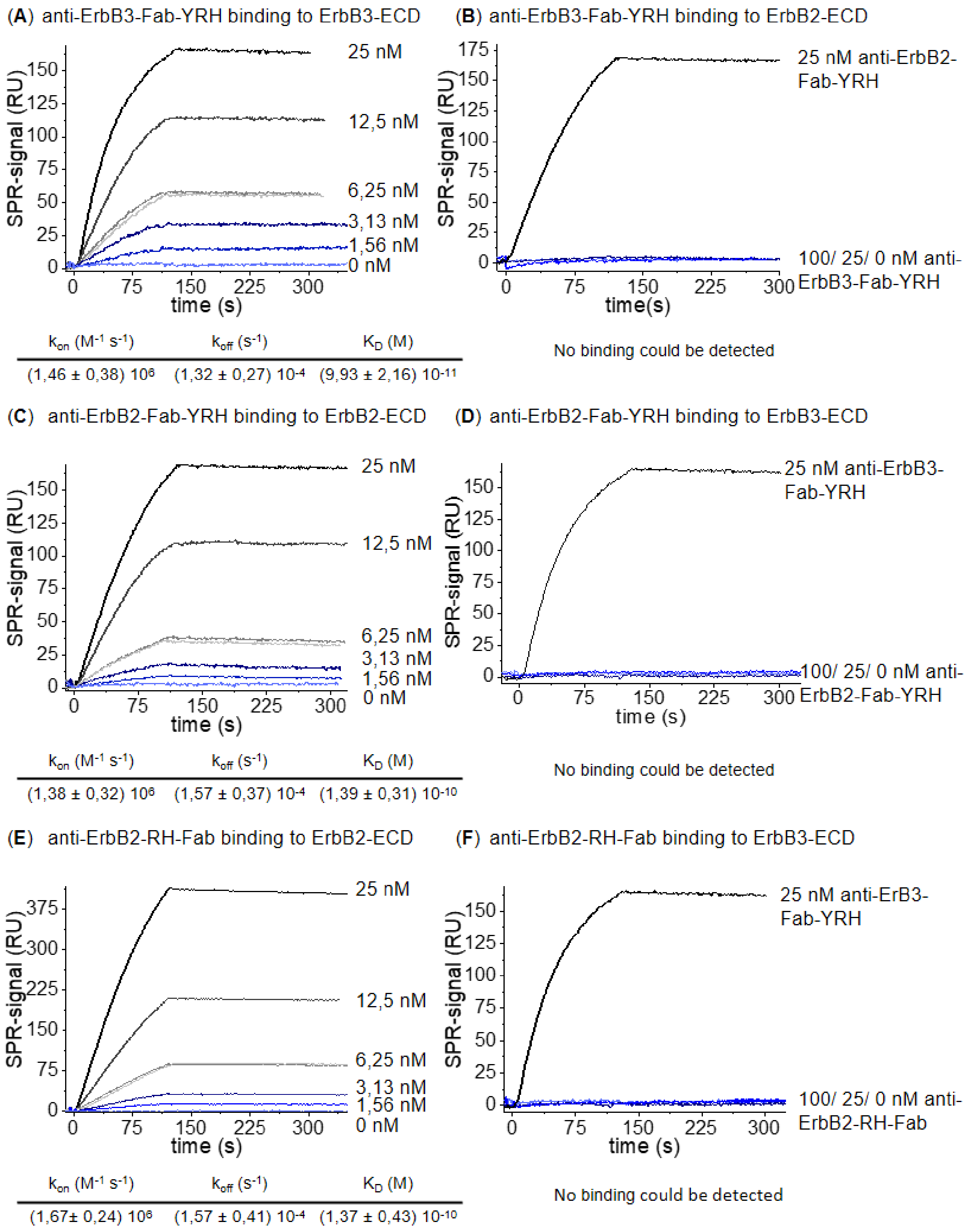

Figure S6: SPR-sensograms, k_{on}/k_{off} and K_D values of the binding of the monospecific Fabs to the ErbB2 and ErbB3 ectodomains. Anti-ErbB3-Fab-YRH binding to ErbB3 ectodomain (A) and to ErbB2 ectodomain (B); anti-ErbB2-Fab-YRH binding to ErbB2 ectodomain (C) and to ErbB3 ectodomain (D); anti-ErbB2-RH-Fab binding to ErbB2 ectodomain (E) and to ErbB3 ectodomain (F); ECD: ectodomain

Reaction conditions: 120 s association, 180 s dissociation in PBS, pH 7.4, flow rate: 30 µl/min, 25 °C

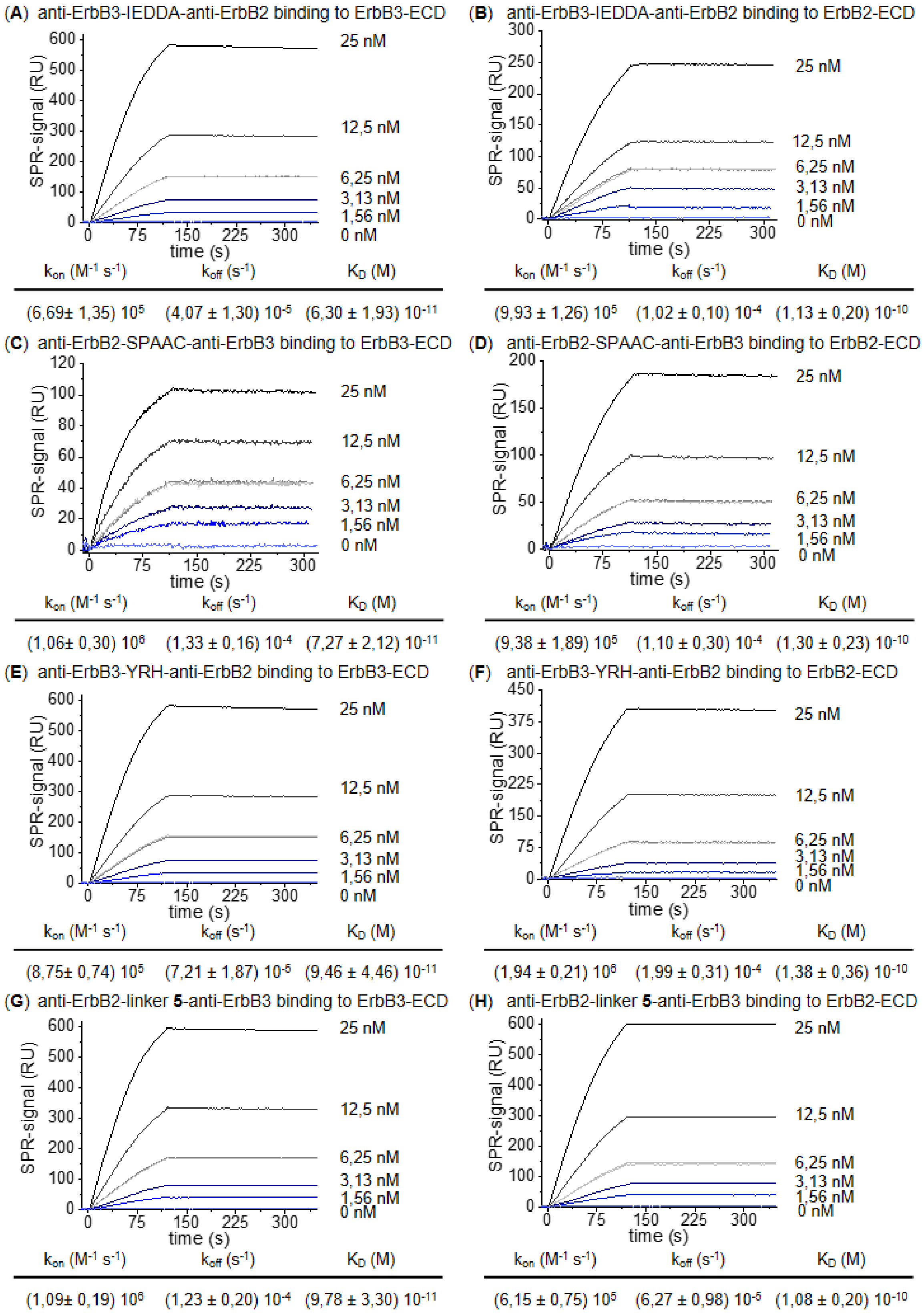

Figure S7: SPR-sensograms, k_{on}/k_{off} and K_D values of the binding of the bispecific bsFabs to the ErbB2 and ErbB3 ectodomains. Anti-ErbB3-Fab-IEDDA-anti-ErbB2-bsFab binding to ErbB3 ectodomain (A) and to ErbB2 ectodomain (B); anti-ErbB2-SPAAC-anti-ErbB3-bsFab binding to ErbB3 ectodomain (C) and to ErbB2 ectodomain (D); anti-ErbB3-YRH-anti-ErbB2-bsFab binding to ErbB3 ectodomain (E) and to ErbB2 ectodomain (F); anti-ErbB2-linker **5**-anti-ErbB3-bsFab binding to ErbB3 ectodomain (G) and to ErbB2 ectodomain (H); ECD: ectodomain
Reaction conditions: 120 s association, 180 s dissociation in PBS, pH 7.4, flow rate: 30 µl/min, 25 °C

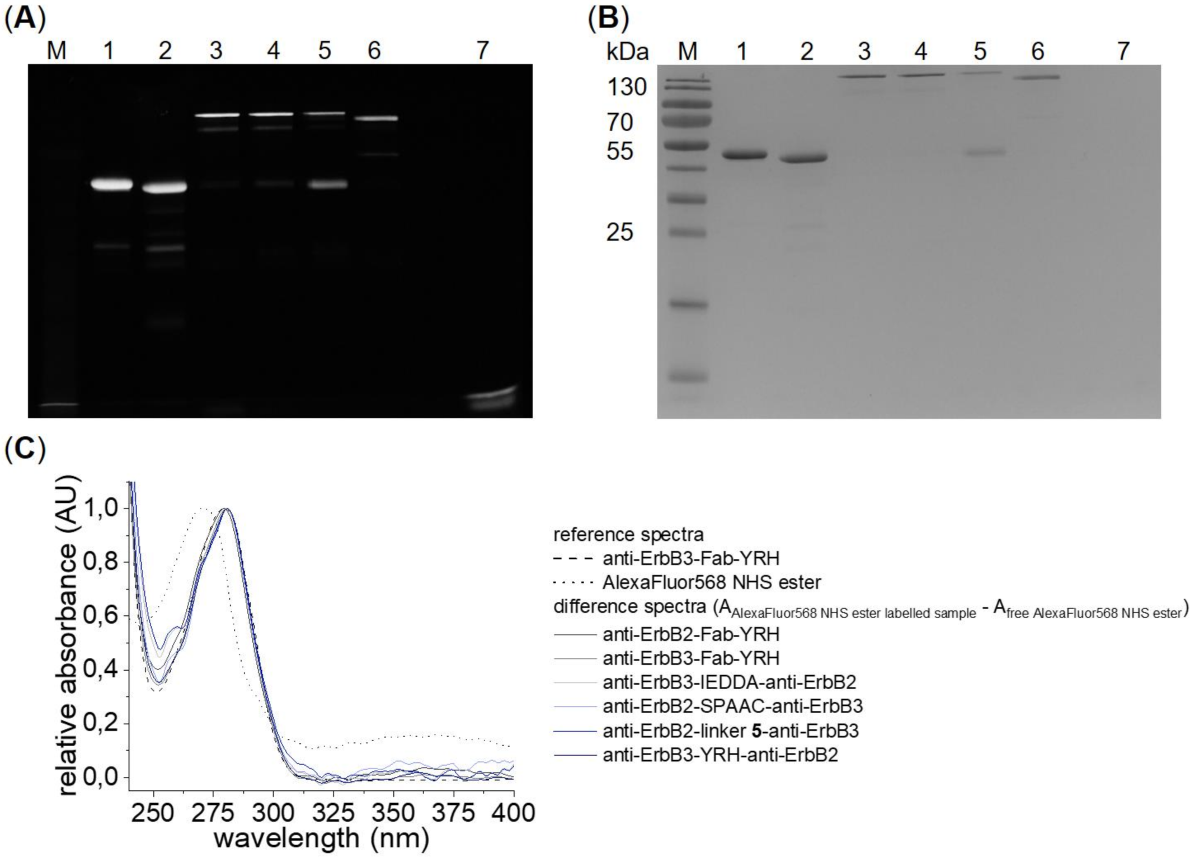

Figure S8: Analysis of dye-induced aggregation of Fabs and bsFabs after AlexaFluor568 modification. Analysis based on non-reducing SDS-PAGE detected by fluorescence (A) and Coomassie staining (B). Note that there are no additional migration bands at higher molecular weight indicating aggregation, 1: anti-ErbB2-Fab-YRH, 2: anti-ErbB3-Fab-YRH, 3: anti-ErbB3-IEDDA-anti-ErbB2 bsFab, 4: anti-ErbB2-SPAAC-anti-ErbB3 bsFab, 5: anti-ErbB2-linker **5**-anti-ErbB3 bsFab, 6: anti-ErbB3-YRH-anti-ErbB2 bsFab, 7: AlexaFluor568 NHS ester, M: molecular marker; (C) UV/Vis spectroscopic protein analysis in the range of 240-400 nm. The UV/Vis absorbance of the free AlexaFluor 568 NHS dye was subtracted from those of the modified Fabs and bsFabs. Note that neither an additional contribution above 320 nm due to scattering of large particles (e.g. aggregates) nor baseline shifts can be observed. For comparison, spectra of unmodified anti-ErbB3 Fab YRH and AlexaFluor 568 NHS ester are included.

Degree of protein labelling (dye/protein): anti-ErbB2-Fab-YRH: 11.7, anti-ErbB3-Fab-YRH: 11.3, anti-ErbB3-IEDDA-anti-ErbB2 bsFab: 3.2, anti-ErbB2-SPAAC-anti-ErbB3 bsFab: 4.2, anti-ErbB2-linker **5**-anti-ErbB3 bsFab: 3.1, anti-ErbB3-YRH-anti-ErbB2 bsFab: 2.3.

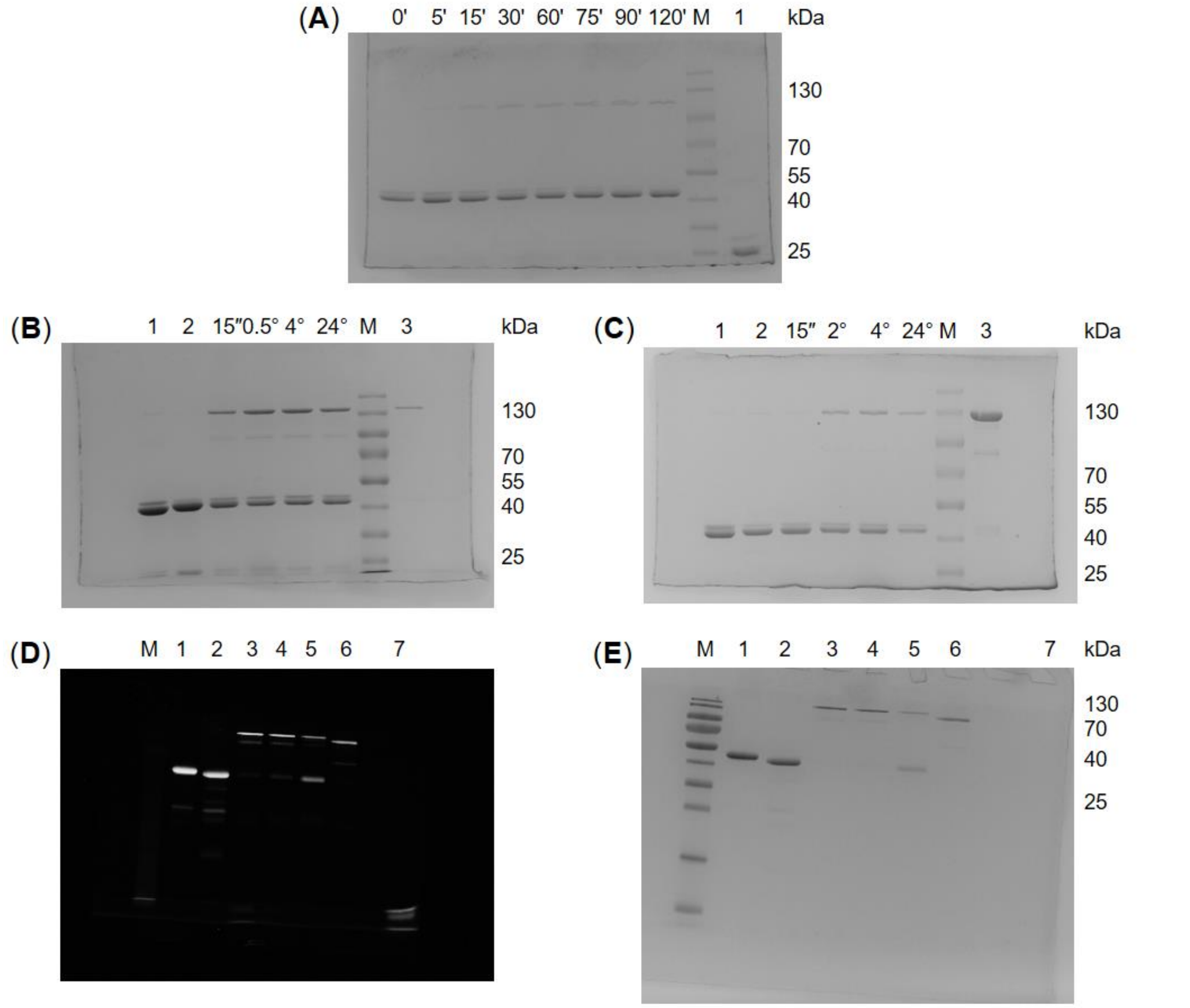

Figure S9: Original images of the SDS-PAGE analyses presented in the main section and supplementary materials of the paper. (A) Original image belonging to Figure 2B, 1: reduced sample after 60 min, M: molecular marker; (B) Original image belonging to Figure 4C, 1: anti-ErbB3-Fab-MeTz, 2: anti-ErbB2-Fab-TCO, 3: purified anti-ErbB3-IEDDA-anti-ErbB2-bsFab, M: molecular marker; (C) Original image belonging to Figure S1D, 1: anti-ErbB2-Fab-DBCO, 2: anti-ErbB3-Fab-PAA, 3: purified anti-ErbB2-SPAAC-anti-ErbB3-bsFab, M: molecular marker, (D) Original image belonging to Figure S8A, 1: anti-ErbB2-Fab-YRH, 2: anti-ErbB3-Fab-YRH, 3: anti-ErbB3-IEDDA-anti-ErbB2-bsFab, 4: anti-ErbB2-SPAAC-anti-ErbB3-bsFab, 5: anti-ErbB2-linker **5**-anti-ErbB3-bsFab, 6: anti-ErbB3-YRH-anti-ErbB2-bsFab, 7: AlexaFluor568 NHS ester, M: molecular marker; (E) Original image belonging to Figure S8B, 1: anti-ErbB2-Fab-YRH, 2: anti-ErbB3-Fab-YRH, 3: anti-ErbB3-IEDDA-anti-ErbB2-bsFab, 4: anti-ErbB2-SPAAC-anti-ErbB3-bsFab, 5: anti-ErbB2-linker **5**-anti-ErbB3-bsFab, 6: anti-ErbB3-YRH-anti-ErbB2-bsFab, 7: AlexaFluor568 NHS ester, M: molecular marker
